# T and N Staging of Gastric Cancer Using Dual-Source Computed Tomography

**DOI:** 10.1155/2018/5015202

**Published:** 2018-12-04

**Authors:** Zhao-Yong Xie, Rui-Mei Chai, Guo-Cheng Ding, Yi Liu, Ke Ren

**Affiliations:** ^1^Department of Radiology, The First Affiliated Hospital, China Medical University, Shenyang, 110001 Liaoning Province, China; ^2^CT and MRI Section, Chifeng City Hospital, Chifeng, 024000 Inner Mongolia, China

## Abstract

**Aim:**

This study is aimed at comparing gastric cancer T and N staging between virtual monochromatic energy images and fusion images generated by dual-source computed tomography (DSCT) dual-energy mode data acquisition prospectively while measuring the iodine concentration of gastric cancer and lymph nodes at different T and N stages from iodine map retrospectively.

**Methods:**

A total of 71 patients (50 males and 21 females; mean age: 59 ± 11 years) confirmed with gastric cancer by endoscopic biopsy with no neoadjuvant chemotherapy were enrolled for the CT examination before surgeries. The preoperative T and N staging results were compared between groups with pathological results as the gold standard. The iodine concentrations of the gastric lesions and LNs were measured on the iodine-based material decomposition images. All iodine concentration values were normalized against those in the abdominal aorta and defined as normalized iodine concentration (nIC) values. The short axis length of LNs and nIC values were statistically analyzed.

**Results:**

Group A was better than group B for T3 and T4 staging. No statistically significant difference in the overall accuracies for N staging was found between groups. For the late arterial and delayed phases, T3 and T4 nIC values of the extraserosal adipose tissue showed statistically significant differences. The nIC values between N0 and Nm (N1–N3) showed statistically significant differences in the portal phase only.

**Conclusions:**

T3 and T4 nIC values of the extraserosal adipose tissue showed statistically significant differences. Hence, dual-source CT may be helpful in the differential diagnosis between T3 and T4.

## 1. Introduction

Gastric cancer is one of the most common cancers worldwide. According to the report of GLOBOCAN 2012 from the International Agency for Research on Cancer, 952,000 new gastric cancer cases and 723,000 deaths due to gastric cancer were reported all over the world in 2012 [1]. Siegel et al. [2] reported that the number of new cases of gastric cancer was 21,600 and the number of deaths was 10,990 in the United States. Consequently, a large number of preoperative staging studies on gastric cancer were performed using multidetector row computed tomography (MDCT) [3]. Compared with endoscopic ultrasonography (EUS), computed tomography (CT) is more valuable for TNM staging of gastric cancer [4, 5]. In the meantime, the performance of positron emission tomography (PET)/CT and functional magnetic resonance imaging (MRI) techniques needs to be further studied [6]. In fact, the prognosis of patients depends on the clinical stage of cancer [7], including the invasion depth to the gastric wall, LN metastasis, and distant metastasis. Of these three aspects, the metastasis of LN is crucial. However, abdominal ultrasonography (AUS), EUS, MDCT, conventional MRI, and FDG-PET cannot be used to confirm or exclude LN metastasis reliably [8]. Moreover, the successful introduction of neoadjuvant chemotherapy in treating locally advanced gastric cancer is crucial in the preoperative imaging identification of responding patients to customize treatment and reduce health care costs [9–11]. Pan et al. [12], Chen et al. [13], and Meng et al. [14] investigated gastric cancer using dual-energy spectral CT (DEsCT) and spectral CT. However, a few studies on gastric cancer used dual-source CT (DSCT). The present study focused on T and N staging of gastric cancer using dual-energy mode DSCT.

## 2. Materials and Methods

### 2.1. Patients

This study was approved by the Hospital Ethics Committee. From July 2013 to December 2013, 76 patients with gastric cancer confirmed by biopsy with no neoadjuvant chemotherapy agreed to undergo dual-source dual-energy mode CT (Definition Flash, Siemens Healthcare, Forchheim, Germany) imaging before surgeries. Five patients underwent palliative resection because of the adjacent organ invasion and multiple para-aortic lymph nodes involvement. LNs were removed as much as possible in the five surgeries. Five patients refused to undergo surgery because of distant metastasis. Thus, 71 patients (50 males and 21 females, mean age: 59 ± 11 years) were enrolled for the study. Lesions were distributed as follows: 18 in the gastric body, 32 in the gastric antrum, 4 in the proventriculus, and 6 with multiple sites of involvement. All patients were diagnosed with adenocarcinoma. Gastric cancers were classified into differentiated and undifferentiated types [9]. Tubular and papillary adenocarcinomas were considered to be differentiated, while poorly differentiated lesions and signet ring cell carcinoma were considered to be undifferentiated. Mucinous carcinoma full of papillary and tubular structures was considered to be differentiated. Mucinous carcinoma with lots of signet ring cells was considered to be undifferentiated. T and N staging was done according to the criteria of AJCC and UICC (7th UICC TNM Staging System of Gastric Cancer).

### 2.2. CT Scan

Patients with 6 h fasting drank 800–1000 mL of tap water to achieve gastric distension just before CT scan. All patients were placed in a supine position, and the CT scan parameters were set as follows: 32 × 0.6 mm collimation, pitch 0.9, rotation time 0.5 s, B30f reconstruction algorithm, and fusion coefficient of 0.5. The online tube current modulation of CARE Dose 4D (Siemens Healthcare, Forchheim, Germany) was switched on, and tube voltages were set as 100 kV and 140 kV. A total of 70 mL of contrast agent of iohexol (350 mgI/mL) and 30 mL of 0.9% saline were injected using a high-pressure syringe at a rate of 2.5 mL/s to 3 mL/s. Triphasic CT scans were taken during the late arterial phase (start of delay, 40 s), portal phase (70 s), and delayed phase (150 s). The entire abdomen was examined. The datasets were reconstructed with 1 mm slice thickness and transferred to a dedicated workstation with dual-energy software (syngo.via, Siemens Healthcare, Forchheim, Germany) for analysis.

### 2.3. CT Staging

**Figure 1:**
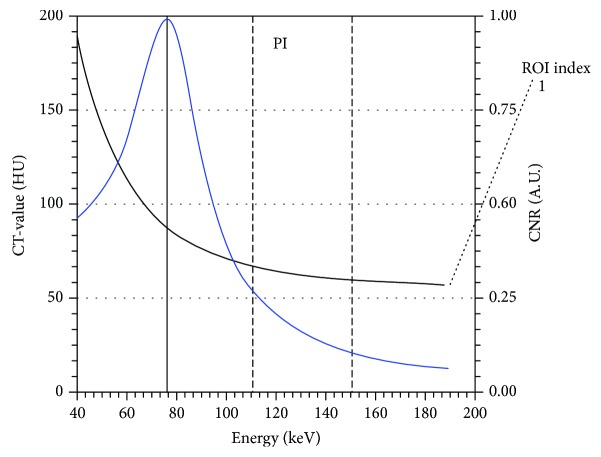
From 40 keV to 190 keV, the blue line represents the change in CNR. The white line represents the CT attenuation change in ROI. CNR was 1.0 when keV was set to be 76.

The monochromatic images (CNR = 1) were reconstructed as images in group A using a workstation, as illustrated in Figure 1, and the fused images of group B had a merging index of 0.5. Two radiologists specialized in gastrointestinal imaging with more than 5-year experience were blinded to the endoscopic results while offering the T and N staging from multiplanar reconstruction (MPR) of 1 mm slice thickness images of group A and group B independently.

The criteria for defining T staging of MDCT was that proposed by Kim et al. [15]: T0, no abnormal findings of the gastric wall with normal fat plane; T1a, tumor showing enhancement and/or thickening of the inner mucosal layer compared with the adjacent normal mucosal layer, with an intact low-density-stripe layer; T1b, disruption of the low-density-stripe layer (less than 50% of the thickness); T2, disruption of the low-density-stripe layer (greater than 50% of the thickness) without abutting on the outer, slightly high-attenuating layer; T3, discrimination between the enhancing gastric lesion and the outer layer visually impossible and a smooth margin of the outer layer or a few small linear strandings in the perigastric fat plane; T4a, an irregular or nodular margin of the outer layer and/or a dense band-like perigastric fat infiltration; and T4b, obliteration of the fat plane between the gastric lesion and the adjacent organs or direct invasion of the adjacent organs. Metastatic lymph nodes were diagnosed using CT based on the following criteria: short axis length of LNs around the stomach greater than 6 mm and the ones far from the stomach with a short axis length greater than 8 mm. In addition, the central necrotic LNs and clustered nodes (three or more than three nodes) around the stomach regardless of node size were considered to be local metastasis. According to the 7th UICC TNM Staging System of Gastric Cancer, N staging criteria for MDCT was defined as follows: N0, no lymph nodes involved; N1, one or two lymph nodes involved; N2, three to six lymph nodes involved; N3a, seven to 15 lymph nodes involved; N3b, more than 16 lymph nodes involved.

### 2.4. Measurement of CT Attenuation

**Figure 2:**
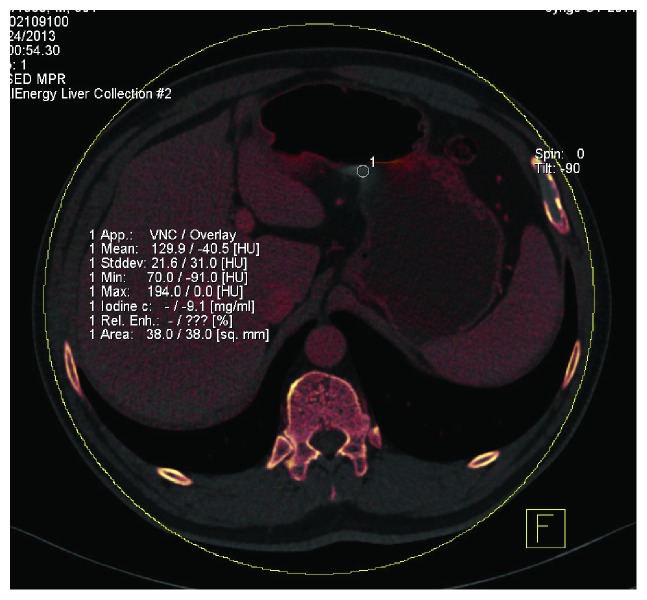
Measurement of the motion artifact could not reflect the real status. It was −9.1 mg/m.

**Figure 3:**
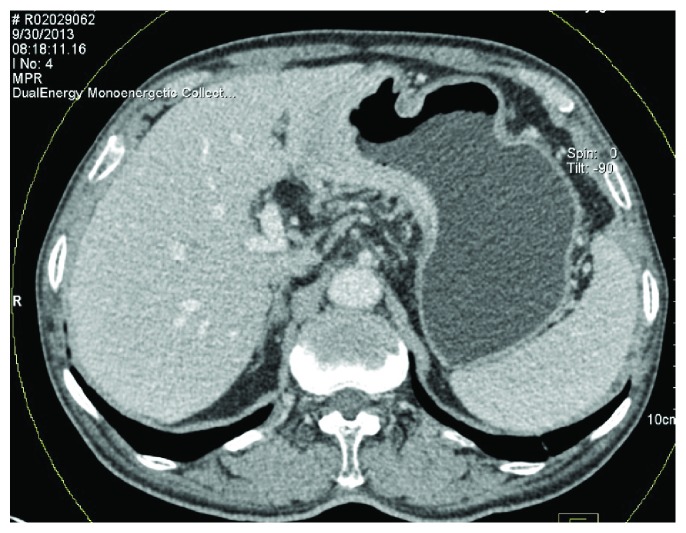
When the monoenergy value was set to be 75 keV with CNR equaled to 1.0, a large number of soft tissue strands stretched from the serous surface were seen.

**Figure 4:**
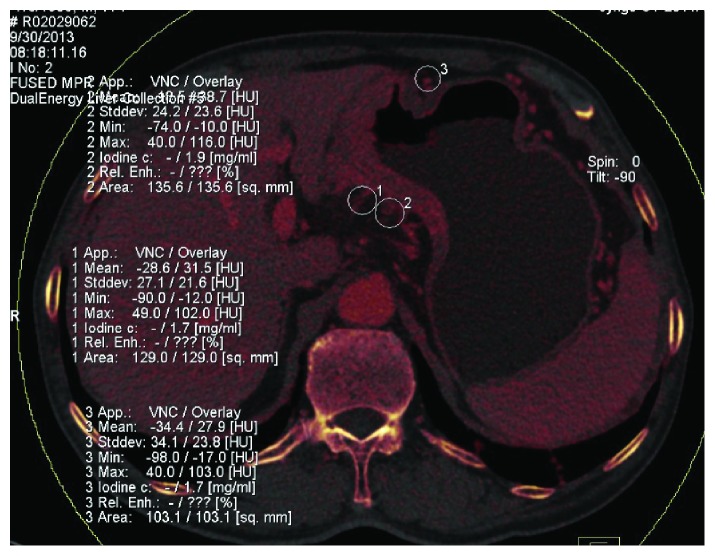
Measurement of the extraserosal adipose tissue in a 77-year-old male patient staged as T3N3bM0 (same patient as in Figure 3).

With surgical and histological findings as reference, the location of gastric cancer lesions, the status of metastatic and nonmetastatic LNs, and CT enhancement from iodine map were evaluated. The iodine concentrations in lesions and lymph nodes were normalized as proposed by Pan et al. [12] (nIC = IC_lesion_/IC_aorta_) to minimize the variation among the three enhanced phases and individualized patients. IC_lesion_ was for the iodine concentration of the region of interest (ROI); IC_aorta_ was for the iodine concentration of the abdominal aorta. The gastric wall lesions were located using MPR images of group A and then analyzed using liver virtual noncontrast (VNC) (Siemens Healthcare), which could generate the virtual noncontrast images and the iodine map. Artifacts caused by gastric peristalsis must be avoided, as shown in Figure 2. ROI should be as large as possible and be measured for two to three continuous layers in the axial or MPR images. Three to five ROIs were measured for the same gastric lesion. T3 and T4 nIC values of the extraserosal adipose tissue, which was adjacent to gastric lesions, were measured using MPR images, with circular ROI (100–150 mm^2^) against the gastric serosa and avoiding the adjacent organs, as shown in Figures 3 and 4. Small LNs, fine blood vessels, and fibrous bands were included. For LN measurement, only values of N0 patients confirmed by surgery were recorded as the values of nonmetastatic LNs. For LNs that could not be detected using CT, the short axis lengths were defined as 0.4 mm. For LNs with a short axis length greater than 15 mm, ROIs in the center and near the edge were measured. Disagreement on measurement between two radiologists was resolved by consensus.

### 2.5. Statistical Analysis

The accuracy, sensitivity, and specificity of gastric cancer T and N staging for groups A and B were calculated. nIC values of both cancer lesions and LNs were calculated and classified by different T and N staging. The nIC values of T3 and T4 in the extraserosal adipose tissue were calculated and compared. The chi-square test, independent-samples *t* test, and analysis of variance were used in this study. nIC values were expressed as mean ± standard deviation (SD). All data were analyzed using the SPSS 13.0 software (SPSS Inc., IL, USA). A *P* value less than 0.05 indicated statistical significance.

## 3. Results

The mean age of the 71 patients included in this study was 59 ± 11 years. Of all the gastric lesions, 18 were located in the gastric body; 32 in the gastric antrum; 9 in the stomach cardia; 4 in the stomach pylorus; and 6 with multiple sites of involvement. The average keV for group A was 75 ± 1 keV.

### 3.1. Preoperative T and N Staging Results of Groups A and B

The preoperative T and N staging results of groups A and B are shown in Tables 1 and 2. The overall accuracy for T staging was 73.23% and 60.56% and the overall accuracy for N staging was 70.4% and 64.8% for group A and group B, respectively. Group A was better than group B (*P* < 0.05), especially for T3 and T4 staging (*P* = 0.004, *P* < 0.05). However, no statistically significant difference was found between T1 and T2 stages. The overall accuracies for N staging showed no statistically significant differences between the two groups (*P* = 0.125).

### 3.2. Statistical Analysis of nIC Values after Surgeries

#### 3.2.1. Measurement of nIC of Different T Stages of Lesions

Table 3 shows nIC values of different T stages of lesions in different phases. nIC values of gastric lesions in the late arterial phase, portal phase, and delayed phase were 0.19 ± 0.14, 0.49 ± 0.24, and 0.65 ± 0.29, respectively. nIC values between the three enhanced phases showed statistically significant differences (*P* < 0.05). However, no statistically significant differences in nIC values in all phases were found between differentiated and undifferentiated cancers (*P* > 0.05). For differentiated cancer, nIC values in the late arterial phase showed statistically significant differences between the portal and delayed phases (*P* < 0.05), whereas no statistically significant differences were shown between the portal and delayed phases (*P* > 0.05). For undifferentiated cancer, nIC values of gastric lesions showed statistically significant differences among all the enhanced phases (*P* < 0.05).

#### 3.2.2. Short Axis Length and nIC Values of LN

A total of 2186 LNs were detected by histopathology. Of these, 339 were diagnosed as metastatic LN. Using CT examinations, 51 LNs were measured from 25 N0 patients, 24 LNs were measured from 15 N1 patients, and 64 and 251 LNs were measured from 16 N2 patients and 15 N3 patients, respectively. Further, 24% (82/339) metastatic LNs were not found by radiologists in CT images. For N staging, the general accuracy of groups A and B was 70.4% and 64.8%, respectively. No statistically significant difference was observed between the two groups. For the short axis length of LNs in Table 4, no statistically significant differences between N0 and N1 and N1 and N2 were shown, while statistically significant differences were noted between the remaining N stages (*P* < 0.05). Of all the enhanced phases, nIC values between N0 and other N stages showed statistically significant differences in the portal phase only (*P* < 0.05).

### 3.3. Radiation Dosimetric Evaluation

The average effective dose (ED) of this study was 12.50 mSv. Average ED for unenhanced sequence, the late arterial phase, portal phase, and delayed phase was 3.08 mSv, 3.11 mSv, 3.14 mSv, and 3.18 mSv, respectively.

## 4. Discussion

Dual-energy CT is an increasingly used technology for some clinical indications [16–19]. In the present study, two radiologists of two groups performed the T and N staging before the surgery without knowing the results of gastroscopy. However, they knew that they were part of a study on gastric cancer. Hence, the T and N staging method used in the present study was partially blind. Similar to this study, many other studies did not include patients without gastric cancer as the control group [3, 12, 15, 20]. Kim et al. [21] and Park et al. [22] used partially and completely blind methods in their gastric cancer studies and showed no difference in the two methods. Hence, the present study was not affected by the lack of a negative contrast group. MPR techniques can improve the differential diagnosis between T3 and T4 stages of gastric cancer [23]. MPR techniques were implemented by the two radiologists with the same workstation in this study, and the slice thickness was set to be 1 mm. The overall accuracy for groups A and B was 73.23% and 60.56%, respectively. The accuracy of T1, T2, T3, and T4 staging for groups A and B was 91.5%, 83.1%, 83.1%, and 88.7% and 91.5%, 83.1%, 69%, and 77.5%, respectively. The sensitivity of T1, T2, T3, and T4 staging for groups A and B was 63.6%, 50%, 76.5%, and 86.2% and 54.5%, 57.1%, 47.1%, and 72.4%, respectively. The specificity of T1, T2, T3, and T4 staging for groups A and B was 96.7%, 91.2%, 87.5%, and 90.5% and 98.3%, 89.5%, 75.9%, and 81%, respectively. In terms of accuracy, groups A and B showed a statistically significant difference (*P* < 0.05), as revealed using McNemar's test. Group A was better than group B. For T3 and T4 staging, McNemar's test results showed a statistically significant difference (*P* < 0.05) between the two groups; group A was better than group B. However, no statistically significant difference was observed between T1 and T2 staging. Nie et al. [8] reported that EUS might be superior to MDCT in preoperative T1 and N staging. The results of T and N staging indicated that single keV images with CNR equaled to 1 helped in the differential diagnosis of T3 and T4 staging.

**Figure 5:**
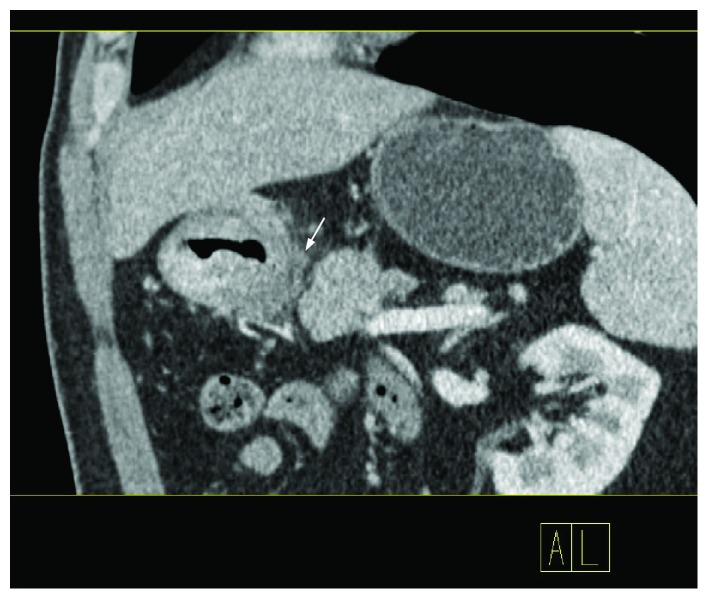
In a 45-year-old male patient, MPR image of 75 keV showed poorly differentiated antrum cancer (T4aN1). The white arrow shows the serous surface invasion of the stomach. The space between the stomach and the pancreas was clear.

**Figure 6:**
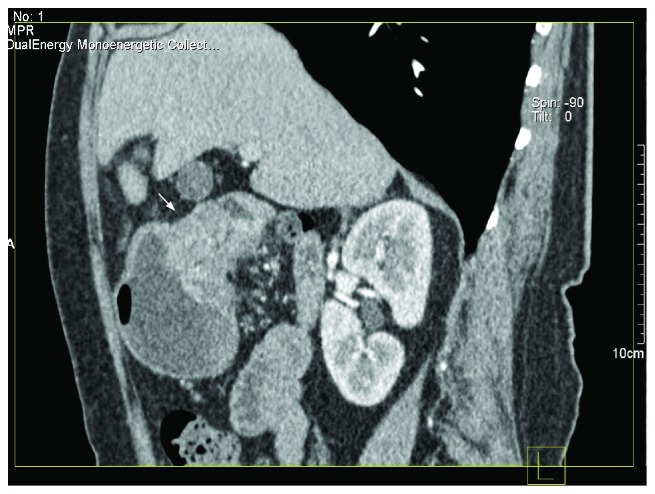
In a 74-year-old female patient, MPR image of 75 keV showed highly differentiated antrum cancer (T3N2). The serous surface was clear.

nIC values of T3 and T4 extraserosal adipose tissue showed statistically significant differences in the late arterial and delayed phases (*P* < 0.05), while no statistically significant difference was observed in the portal phase. This was different from the findings of Meng et al. [14]. The results indicated that T4 extraserosal adipose tissue was invaded by gastric cancer. Vessels and lymph tissues increased in the extraserosal adipose tissue because of invasion. The macroscopic diagnosis of serosal invasion was largely consistent with pathological findings [24]. The nIC values detected using spectral CT correlated with the microvessel density (MVD). nIC-AP and nIC-VP could reflect angiogenesis in different pathological subgroups of advanced gastric cancer [13]. Also, T4 extraserosal adipose tissue was enhanced in the late arterial phase and showed a delayed enhancement as gastric cancer lesion itself [25]. A precise T3 and T4 staging before the surgery has clinical significance. The main mode of metastasis of advanced gastric cancer is serosal invasion; it is an important cause of cancerous ascites [26], recurrence, and death [27, 28]. Single keV images with CNR equaled to 1 and 1 mm MPR images led to much clearer vision in the extraserosal adipose tissue.  Figure 5 from group A shows clear serosal invasion and signs of pancreatic invasion. The sensitivity of both groups increased from T1 to T4 stage. This indicated that the invasion depth and width of the gastric wall were independent factors [22].  Figure 6 shows a clear serosal surface in a patient with highly differentiated gastric adenocarcinoma staged as T3N2.

A total of 71 patients with gastric cancer were sorted as differentiated and undifferentiated cancer as in the study by Kim et al. [29]. For nIC values, no statistically significant differences were found between differentiated and undifferentiated cancer in the three enhanced phases. This was different from the results of Pan et al. [12], which showed statistically significant differences in nIC values between the differentiated and undifferentiated cancer in the late arterial and portal phases. The difference between two studies might be related to different contrast media injection protocols. In this study, there was no correlation between body mass index (BMI) and contrast agent injected. Statistically significant differences in nIC values were found between the enhanced phases. No statistically significant differences in nIC values of differentiated cancer were found between the portal and delayed phases. Statistically significant differences in nIC values of undifferentiated cancer were found between the enhanced phases (*P* < 0.05). Therefore, further studies are needed to explore the relationship between the nIC values of different pathological types.

A large number of studies using CT focused on the LNs of gastric cancer. However, no unified standard has been established for the diagnosis of metastatic LNs using CT. Some of the following criteria were applied: perigastric LNs with short axis diameter of 6 mm and LNs far from the stomach larger than 8 mm in diameter [20], short axis diameter larger than 8 mm and an oval shape [30], and LNs larger than 10 mm or 7 to 10 mm circular LNs with obvious enhancement [31]. Lymph node metastasis is related to the treatment of early and advanced gastric cancer. It is one of the most important postoperative prognostic factors. D2 radical gastrectomy is the gold standard of surgery for advanced gastric cancer. However, in specialized centers, super extended (D3) lymphadenectomy, which includes systematic removal of posterior stations, allows a better control of the disease with a lower incidence of locoregional relapse compared to the standard D2 dissection. New surgical treatments are available for early gastric cancer; the choice depends on the state of LN metastasis and the range [32, 33]. Zhang et al. [34] set a support vector machine (SVM) model based on MDCT. They investigated LN metastasis of gastric cancer, with serosal invasion, tumor grade, tumor maximal size, number of LNs, and the longest diameter of LNs and station of LNs representing the biological behavior of gastric cancer. They believed that the SVM model could help predict lymph node metastasis preoperatively. Zhang et al. [34] reported that the ratio between negative and positive lymph nodes could help evaluate the postoperative prognosis and the number of metastatic and nonmetastatic LNs. In the present study, N staging of two groups was performed qualitatively before the surgery and the nIC values of LNs were measured to specify the state of LNs.

Table 2 shows the N staging results before the surgery and histological results after the surgery. The overall accuracy of groups A and B was 70.4% and 64.8%, respectively, higher than the findings of Kim et al. (63.2%) [29], who used 3 mm thick images. The thickness of images might be the reason for the difference in the findings of the two studies. In the present study, McNemar's test results showed no statistically significant difference between groups. Single keV images of group A with CNR equaled to 1 and fusion images of group B showed no difference in N staging.

Table 4 shows short axis length and nIC values of LNs in different N stages. It was hard to sort the N stages by short axis length only. It was concluded that the more the LNs detected by CT and the longer the short axis length, the more the chances to be invaded by cancer. Morgagni et al. [35] reported that the involvement of LNs could not be predicted. In the present study, statistically significant differences were observed in nIC values of N0 LNs and LNs involved in the portal phase only (*P* < 0.05). No statistically significant difference was observed between LNs from differentiated and undifferentiated cancers in all enhanced phases. This indicated that it was impossible to predict the histological type of gastric cancer by the nIC values of involved LNs. However, Tawfik et al. [36] reported in their study on neck LNs that a differential diagnosis could be made between malignant and inflammatory LNs by the threshold nIC value 2.8 mg/mL.

For radiation dosimetric evaluation, the average effective dose (ED) of this study was 12.50 mSv. It was similar to a routine standard CT of the abdomen performed without a dual-energy technique [37]. In this study, nIC values of T3 and T4 extraserosal adipose tissue showed no statistical significant difference in the portal phase. When patients were diagnosed with gastric cancer before scanning, the potential to decrease the overall radiation exposure to patients by eliminating the routine acquisition of unenhanced data and portal phase data was a major benefit of dual-energy CT.

In conclusion, compared with the fusion images, the monochromatic images (CNR = 1) from dual-energy CT performed better in the T staging of gastric cancer, especially for T3 and T4 staging, while no statistically significant differences were found for N staging. The nIC values of the extraserosal adipose tissue showed statistically significant differences and helped differentiate T3 and T4 in the late arterial and delayed phases.

## Figures and Tables

**Table 1:** Preoperative T staging and histological results after surgeries.

CT stage		Histological stage				Rate			*P*	
		T0-1 (*n* = 11)	T2 (*n* = 14)	T3 (*n* = 17)	T4 (*n* = 29)	Accuracy (%)	Sensitivity (%)	Specificity (%)	T3	T4
A	T0-1	7	2	0	0	91.5	63.6	96.7	0.004	0.004
	T2	4	7	0	1	83.1	50.0	91.2		
	T3	0	5	13	3	83.1	76.5	87.5		
	T4	0	0	4	25	88.7	86.2	90.5		
B	T0-1	6	1	0	0	91.5	54.5	98.3		
	T2	5	8	1	0	83.1	57.1	89.5		
	T3	0	5	8	8	69.0	47.1	75.9		
	T4	0	0	8	21	77.5	72.4	81.0		

The overall accuracy of group A was better than that of group B (*P* = 0.004, *P* < 0.05). The T3 and T4 staging showed identical results (*P* = 0.004, *P* < 0.05).

**Table 2:** Preoperative N staging and histological results after surgeries.

CT stage		Histological stage				Rate			*P*
		N0 (*n* = 25)	N1 (*n* = 15)	N2 (*n* = 16)	N3 (*n* = 15)	Accuracy (%)	Sensitivity (%)	Specificity (%)	
A	N0	20	4	0	0	87.3	80.0	91.3	0.125
	N1	3	9	4	1	80.3	60.0	85.7	
	N2	2	2	9	2	81.7	56.3	89.1	
	N3	0	0	3	12	91.5	80.0	94.6	
B	N0	18	4	0	0	84.5	72.0	91.3	
	N1	5	9	5	1	76.1	60.0	80.4	
	N2	2	2	8	3	78.9	50.0	87.3	
	N3	0	0	3	11	90.1	73.3	94.6	

No statistically significant differences in overall accuracies were found between groups A and B (*P* = 0.125).

**Table 3:** nIC values of different T stages and lesions.

Lesions	*n*	nIC-A	*P*	nIC-P	*P*	nIC-D	*P*
T1	11	0.14 ± 0.07	0.09	0.48 ± 0.30	0.08	0.82 ± 0.21	0.09
T2	14	0.25 ± 0.15		0.45 ± 0.27		0.59 ± 0.41	
T3	17	0.13 ± 0.06		0.45 ± 0.13		0.60 ± 0.23	
T4	29	0.21 ± 0.17		0.55 ± 0.26		0.64 ± 0.26	
Differentiated cancer	35	0.21 ± 0.16	0.06	0.48 ± 0.21	0.07	0.56 ± 0.24	0.09
Undifferentiated cancer	36	0.17 ± 0.11		0.51 ± 0.27		0.73 ± 0.31	
T3 extraserosal adipose tissue	17	0.08 ± 0.05	0.004	0.23 ± 0.13	0.06	0.25 ± 0.13	0.001
T4 extraserosal adipose tissue	29	0.10 ± 0.06		0.29 ± 0.16		0.40 ± 0.32	

nIC-A, nIC-P, and nIC-D represented the nIC values of late arterial phase, portal phase, and delayed phase, respectively. Of all the enhanced phases, nIC values among different T stages showed no statistically significant differences (*P* > 0.05). nIC values between differentiated and undifferentiated cancers showed no statistically significant differences (*P* > 0.05). However, nIC values of the extraserosal adipose tissue between T3 and T4 showed statistically significant differences in both late arterial phase and delayed phase (*P* = 0.004 and *P* = 0.001, *P* < 0.05).

**Table 4:** Short axis length and nIC values of LN in different N stages.

	*N*_P_	*N*_LN_	Short axis length (cm)	nIC-A	nIC-P	nIC-D
			$\bar{\chi }\pm SD$	$\bar{\chi }\pm SD$	$\bar{\chi }\pm SD$	$\bar{\chi }\pm SD$
N0	25	51	0.48 ± 0.07	0.18 ± 0.10	0.56 ± 0.39	0.64 ± 0.30
N1	15	24	0.58 ± 0.26	0.20 ± 0.05	0.44 ± 0.08	0.50 ± 0.10
N2	16	64	0.66 ± 0.27	0.12 ± 0.09	0.31 ± 0.16	0.37 ± 0.17
N3	15	251	1.1 ± 0.64	0.16 ± 0.09	0.35 ± 0.13	0.49 ± 0.23
N0	25	51	0.51 ± 0.10	0.18 ± 0.10	0.56 ± 0.39	0.64 ± 0.30
NX	46	339	0.98 ± 0.60	0.15 ± 0.09	0.35 ± 0.14	0.47 ± 0.22

*N*_P_ represents the number of patients. *N*_LN_ represents the number of LNs measured. No statistically significant differences in short axis length were observed between N0 and N1 (*P* > 0.05), while statistically significant differences were found between the remaining N stages (*P* < 0.05). Of all the contrast-enhanced phases, nIC values between N0 and other N stages showed statistically significant differences in the portal phase only.

## Data Availability

The data used to support the findings of this study are included within the article.
